# Perception and Assessment of Psoriasis Among the General Population in the Jazan Region, Saudi Arabia

**DOI:** 10.7759/cureus.48398

**Published:** 2023-11-06

**Authors:** Ahmad H Assiri, Mohammed I Alameer, Mohammed E Mojiri, Zakaria Y Shubayli, Osama A Suwaid, Majed M Qaysi, Ali A Alkaeeal, Abdulrahman S Jathmi, Mofareh S Alraythi, Alhassan H Hobani

**Affiliations:** 1 College of Medicine, Jazan University, Jazan, SAU; 2 Faculty of Medicine, Jazan University, Jazan, SAU

**Keywords:** dermatology, general population, saudi arabia, jazan, assessment, perception, psoriasis

## Abstract

Background: Psoriasis is a worldwide disease. It is a chronic immune-mediated inflammatory disease with cutaneous and systemic symptoms that is characterized by erythematous squamous plaques.

Objectives: To study the perception and assessment of psoriasis among the general population of Jazan, Saudi Arabia.

Materials and methods: This was a cross-sectional study that used a self-administrated electronically distributed questionnaire that enrolled 410 people.

Results: Most participants (79%) were familiar with the term "psoriasis," with family, friends, or acquaintances being the primary source of knowledge. Personal experience with psoriasis was reported by 28.3% of participants, with women being more likely to know someone with the condition. If affected by psoriasis, the majority of respondents would consult a dermatologist (89.3%). Although 48% of participants considered psoriasis to be a significant burden for affected individuals, negative attitudes and discrimination towards those with the condition were prevalent, with a significant proportion stating that they would not want to eat at the same table, shake hands, or be in a personal relationship with someone with psoriasis. Additionally, most participants reported feeling sorry for psoriasis patients but also expressed disgust towards them. Awareness of public actions related to psoriasis was generally low, with only a small percentage of participants familiar with the WHO resolution on psoriasis or World Psoriasis Day.

Conclusion: This study highlights the need for increased education and awareness about psoriasis to address misunderstandings and reduce the burden of the condition on patients. Additionally, efforts are needed to reduce stigma and discrimination towards those with psoriasis, which can significantly impact their quality of life.

## Introduction

Psoriasis is a chronic inflammatory disease with an immune component, clinically presenting with systemic and cutaneous manifestations likely silver erythematous plaques to be the most common presentation [1,2]. Psoriasis was estimated to have a global prevalence of 2-3%, affecting more than 125 million people worldwide, prevalence increases steadily throughout life, also it can manifest at any age [3,4]. The prevalence of psoriasis in Saudi Arabia varies from province to province over time, with the highest reported to be 5.33% in Al-jouf [5]. The etiology of psoriasis, or the cause of the condition, is still not fully understood. However, there are a number of factors that are thought to contribute to the development of psoriasis, including, previous infections, medications, and emotional and physical stress that can also trigger psoriasis [6]. It has also been reported that genetic factors may also be a probable trigger [7,8].

Patients are often stigmatized because psoriatic lesions can be seen in visible locations such as the face, scalp, and hands [9,10]. The stigmatized individuals are more distressed about symptoms and reported greater interpersonal impact and reduced quality of life than their non-stigmatized counterparts. Stigmatization has been reported to be the most significant factor in predicting depression in psoriasis patients [11]. Psoriasis is associated with cardiovascular disease, depressive illness, and psoriatic arthritis [12].

Five types of psoriasis have been reported, including plaque psoriasis, guttate or eruptive psoriasis, inverse psoriasis, pustular psoriasis, and erythrodermic psoriasis [2]. Screening for psoriasis can be conducted by using questionnaire instruments such as the Psoriatic Arthritis Screening and Evaluation and Toronto Psoriatic Arthritis Screening [12,13], while for diagnosis skin biopsy is rarely used, it remains clinical using scoring systems like Psoriasis Area and Severity Index score and Psoriasis Global Assessment score [14,15].

Regarding worldwide studies, a study conducted in Germany and another in Italy found that there is a lack of awareness about psoriasis in the general population. In Germany, 71% of participants were not familiar with the term psoriasis, while 7.3% of participants in Italy were not familiar with it. In both studies, a significant number of participants considered psoriasis to be contagious and had a social stigma enrooted deeply making its management difficult [16,17]. Similarly, a study in Saudi Arabia showed that 78% of the participants were familiar with the term “psoriasis”’ in Arabic and 12.2% considered the disease to be infectious [18]. In an Indian study, the results showed that there was adequate knowledge and overall, 55.84% thought that psoriasis only concerns the skin. Adequate knowledge was directly proportional to a positive attitude [19].

Another study conducted in Tabuk reported that 6% of the participants had the disease. About 63.9% realized that psoriasis is not a contagious disease, but still, 28.8% of participants thought about the probability of being contagious and 7.3% responded with yes it’s contagious. Negative attitudes were also noticed including refusing to share food, shaking hands, and using the same swimming pool [14]. In one more study conducted in the Qassim region, the results showed that 128 (13.5%) respondents had no information about the disease. Social stigma was found to be higher towards psoriatic patients by society [20]. The degree of stigmatization is associated with the level of community and public awareness and knowledge about psoriasis. A multifaceted understanding of the general population regarding psoriasis will encourage advanced efforts to overcome stigma, enhance the quality of life, and improve treatment utilization. Therefore, the present study was conducted to provide insight into the perception, and attitude about psoriasis among the general population in Jazan city, Saudi Arabia. To assess the need for improved health education of the public at large and intend to make recommendations about disease awareness based on the results of the study.

## Materials and methods

Study design

This is an observational cross-sectional study that used a self-administered anonymous electronically distributed questionnaire, aiming to assess the perception of psoriasis among the general population of Jazan, Saudi Arabia.

Sample size

By using the formula ss=(Z2×p×q)/c^2^ the optimal sample size for conducting this study was determined to be 384 participants. Where ss=sample size, Z=1.96, p=0.5, q=(1-p)=0.5, c=sampling error of 5%. In total, 410 participants were enrolled in this study.

Inclusion and exclusion criteria

Participants ≥18 years old, all educational levels, nationalities, and both genders living in the Jazan region were included. We excluded nonresidents of the Jazan region, people with mental impairments, those <18 years of age, and also who are not willing to participate.

Data collection

Data was collected by using a self-administered questionnaire. Each participant was asked to read and sign a consent form, before the start of data collection. The questionnaire was adapted from the validated version generated by the National Conference on Health Care in Psoriasis [16]. The questionnaire was pretested on 10 randomly selected people. The questionnaire consists of 26 close-ended questions. The questionnaire included eight domains: socio-demographic data regarding the participants’ age, sex, nationality, and educational level. The questions used to assess psoriasis perception included questions designed to evaluate disease knowledge and familiarity, personal experience, participants' beliefs about psoriasis, interaction with psoriasis patients, participants’ feelings regarding psoriasis, awareness of public actions, and choice of physician.

Statistical analysis

The data collected was analyzed using Statistical Package for the Social Sciences (IBM SPSS Statistics for Windows, IBM Corp., Version 28.0, Armonk, NY). Data were cleaned and entered into SPSS and analyzed for Descriptive and inferential statistics.

Both the chi-square test and Fisher’s exact test were used to determine the associations between individual categorical variables and the assessment of psoriasis. All categorical variables were reported as percentages. A P-value less than 0.05 was considered significant.

## Results

Demographic data and choice of physician

This study included 410 participants; 167 (40.7%) were men and 243 (59.3%) were women. The age groups included in this study were 18-25 (246; 60%), 26-35 (64; 15.6%), 36-45 (67; 16.3%) and >46 years old (33; 8%). Saudis accounted for 398 (97%) of the participants and non‐Saudis 12 (3%). The majority of the respondents were highly educated (71.7%), having university degrees, with secondary school graduates being less common (22.2%), and those with an educational level of lower than secondary school being the least common (6.1%). More than half of the participants were students (57.6%). If affected by psoriasis or in case of suspected psoriasis, the majority of respondents stated that they would consult a dermatologist (89.3%), followed by a general practitioner (8%). The remaining 2.7% of participants declared that they would consult a physician from another specialty (Table 1).

**Table 1:** Demographic characteristics

Variables	Frequency	Percent
Gender		
Male	167	40.7
Female	243	59.3
Age (years)		
18-25	246	60.0
26-35	64	15.6
36-45	67	16.3
46 and more	33	8.0
Nationality		
Saudi	398	97.1
Non-Saudi	12	2.9
Level of Education		
Below secondary	25	6.1
Secondary	91	22.2
University	294	71.7
State of job		
Students	236	57.6
Not employed	74	18.0
Employe	100	24.4
Income		
Less than 5000 SAR	270	65.9
More than 500 SAR	140	34.1
Marital status		
Married	144	35.1
Single	257	62.7
Widow/Widower	4	1.0
Divorced	5	1.0
Residency		
Urban	208	50.7
Rural	202	49.3
Are you familiar with the term psoriasis?		
Yes	324	79.0
No	86	21.0
If your answer to the previous question is yes what’s your source of information?		
Family, friend's acquaintances	145	35.4
Media	124	30.2
Dermatologist	46	11.2
Family physician	7	1.7
Health insurance information	2	0.5
If affected by psoriasis or in case of suspected psoriasis, to which physician would you consult?		
Consult a dermatologist	366	89.3
General practitioner	33	8.0
Consult a physician from another specialty.	11	2.7

Familiarity with the terms “psoriasis”

Most of the participants (329; 79%) were familiar with the term “psoriasis” in Arabic. This familiarity was significantly associated with participants who were aged above 46 (90% p= 0.043) and also significantly associated with being a female (p=0.001). Among those who claimed to be familiar with the disease, most reported that the source of their knowledge was their family, friends, or acquaintances (35.4%) followed by media (30.2 %), dermatologists (11.2%), family physicians and health insurance information were last by (1.7%) and (0.5%) respectively (Table 2).

**Table 2:** Familiarity with the term psoriasis among the general Jazan population

Variables	Familiarity with the disease		P-value
	Frequency	Percent	
Overall (n=410)	324	79.0	
Gender			
Male (n=167)	114	68.3	<0.001
Female (n=243)	210	86.4	
Age (years)			
18-25 (n=246)	184	74.8	0.043
26-35 (n=64)	52	81.3	
36-45 (n=67)	58	86.6	
46 and more (n=33)	30	90.9	
Nationality			
Saudi (n=398)	314	78.9	0.711
Non-Saudi (n=12)	10	83.3	
Level of Education			
Below secondary (n=25)	18	72.0	0.539
Secondary (n=91)	70	76.9	
University (n=294)	236	80.3	
State of job			
Students (n=236)	177	75.0	0.052
Not employed (n=74)	61	82.4	
Employee (n=100)	86	86.0	
Income			
Less than 5000 SAR (n=270)	210	77.8	0.384
More than 500 SAR (n=140)	114	81.4	
Marital status			
Married (n=144)	123	85.4	0.044
Single (n=257)	194	75.5	
Widow/Widower (n=4)	4	100	
Divorced (n=5)	3	60.0	
Residency			
Urban (n=208)	162	77.9	0.571
Rural (n=202)	162	80.2	

Personal experience with psoriasis

A total of 28.3% of participants stated to know a person with psoriasis; 3.9% were personally affected or had been in the past. Individuals aged between 26 and 35 years, and those with a lower secondary school education and widowers were more strongly affected. On the other hand, women more frequently knew a person with psoriasis than men. The same was true for older (46 and above) compared to younger participants (18-25 years) (Table 3).

**Table 3:** Personal experience with psoriasis

Variables	Know somebody who has psoriasis		Are/have been personally affected by psoriasis		P-value
	Frequency	Percent	Frequency	Percent	
Overall (n=410)	116	28.3	16	3.9	
Gender					
Male (n=167)	39	23.4	9	5.4	0.113
Female (n=243)	77	31.1	7	2.9	
Age (years)					
18-25 (n=246)	54	22.0	5	2.0	<0.001
26-35 (n=64)	17	26.6	6	9.4	
36-45 (n=67)	29	43.3	2	3.0	
46 and more (n=33)	16	48.5	3	9.1	
Nationality					
Saudi (n=398)	113	28.4	15	3.8	0.733
Non-Saudi (n=12)	3	25.0	1	8.3	
Level of Education					
Below secondary (n=25)	7	28.0	2	8.0	0.817
Secondary (n=91)	27	29.7	4	4.4	
University (n=294)	82	27.9	10	3.4	
State of job					
Students (n=236)	53	22.5	7	3.0	0.018
Not employed (n=74)	20	27.0	5	6.8	
Employee (n=100)	43	43.0	4	4.0	
Income					
Less than 5000 SAR (n=270)	65	24.1	9	3.3	0.017
More than 500 SAR (n=140)	51	36.4	7	5.0	
Marital status					
Married (n=144)	54	37.5	8	5.6	0.027
Single (n=257)	59	23.0	7	2.7	
Widow/Widower (n=4)	2	50	1	25.0	
Divorced (n=5)	1	20	0	0	
Residency					
Urban (n=208)	63	30.3	5	2.4	0.227
Rural	53	26.2	11	5.4	

Assessment of psoriasis among the general population

Forty-eight percent of participants considered the disease to be a significant burden for affected individuals; and 29.3% considered it a burden for the relatives of affected individuals. A total of 27.1% thought that effective drugs were available for the treatment of psoriasis; 11% considered psoriasis to be infectious (Table 4).

**Table 4:** Subjective assessment of psoriasis among the general population in the Jazan region

Variables	Psoriasis is a significant burden for affected individuals		Psoriasis is a significant burden for the relatives of affected individuals			Effective drugs are available for psoriasis		Psoriasis is communicable	
	Frequency	Percent		Frequency	Percent	Frequency	Percent	Frequency	Percent
Overall (n=410)	197	48.0		120	29.3	111	27.1	45	11.0
Gender									
Male (n=167)	67	40.1		43	25.7	35	21.0	16	9.6
Female (n=243)	130	53.5		77	31.7	76	31.3	29	11.9
Age (years)									
18-25 (n=246)	104	42.3		55	22.4	53	21.5	19	7.7
26-35 (n=64)	31	48.4		23	35.9	23	35.9	11	17.2
36-45 (n=67)	40	59.7		30	44.8	28	41.9	11	16.4
46 and more (n=33)	22	66.7		12	36.4	7	21.2	4	12.1
Nationality									
Saudi (n=398)	192	48.2		118	29.6	109	27.4	44	11.1
Non-Saudi (n=12)	5	41.7		2	16.7	2	16.7	1	8.3
Level of Education									
Below secondary (n=25)	7	28.0		4	16.0	8	32.0	2	8.0
Secondary (n=91)	39	42.9		22	24.2	24	26.4	8	8.8
University (n=294)	151	51.4		94	32.0	79	26.9	35	11.9
State of job									
Students (n=236)	104	44.1		60	25.4	53	22.5	22	9.3
Not employed (n=74)	35	47.3		20	27.0	23	31.1	6	8.1
Employee (n=100)	58	58.0		40	40.0	35	35.0	17	17.0
Income									
Less than 5000 SAR (n=270)	122	45.2		73	27.0	66	24.4	25	9.3
More than 500 SAR (n=140)	75	53.6		47	33.6	45	32.1	20	14.3
Marital status									
Married (n=144)	78	54.2		55	38.2	46	31.9	21	14.6
Single (n=257)	113	44.0		62	24.1	62	24.1	24	9.3
Widow/Widower (n=4)	4	100		2	50	1	25	0	0
Divorced (n=5)	2	40		1	20	2	40	0	0
Residency									
Urban (n=208)	91	43.8		59	28.4	57	27.4	25	12.0
Rural (n=202)	106	52.5		61	30.2	54	26.7	20	9.9

Interaction with psoriasis patients and participants’ feelings

Regarding interaction with psoriasis patients, 29% of respondents stated that they would not want to eat at the same table as a person with psoriasis; 34.9% stated that they would not shake hands; 14.9% stated that they would not want to live in the same household; 67.1% stated that they would not share the same swimming pool; and 24.6% that they would not want to be in a personal relationship.

Regarding participants’ feelings towards psoriasis, 74.1% of the participants reported that they felt sorry for psoriasis patients, 48% stated that they were disgusted by affected people, and 79.8% thought that patients with psoriasis needed better care for their condition (Table 5).

**Table 5:** Participants’ feelings and interaction with psoriasis patients

Variables	Yes, n (%)	No, n (%)
Feelings regarding psoriasis		
Most people feel sorry for those suffering from psoriasis	304 (74.1)	106 (25.9)
Most people are disgusted by psoriasis	197 (48.0)	213 (52.0)
Most people think that those with psoriasis need better care	327 (79.8)	83 (20.2)
Interaction with Psoriasis Patients		
Eat with psoriasis patient at the same table	291 (71.0)	119 (29.0)
Shake hands with a psoriasis patient	267 (65.1)	143 (34.9)
Use the same swimming pool	135 (32.9)	275 (67.1)
Live in the same household	349 (85.1)	61 (14.9)
Be in a personal relationship with a psoriatic patient	309 (75.4)	101 (24.6)

Awareness of public actions

In assessing the participants’ awareness about the World Health Organization’s (WHO) announcement that psoriasis is considered to be one of five serious noncommunicable diseases, only 77 (18.8%) stated that had heard about the WHO's resolution on psoriasis; Female and middle-aged participants (26-35) also being a Saudi were more familiar. Furthermore, only 66 (16.1%) of respondents were familiar with World Psoriasis Day; being a female and or divorced also aged between 36 and 45)had a great impact (Table 6).

**Table 6:** Awareness of public actions relating to psoriasis in the Jazan region

Variables	WHO resolution		World Psoriasis Day	
	Frequency	Percent	Frequency	Percent
Overall (n=410)	77	18.8	66	16.1
Gender				
Male (n=167)	28	16.8	23	13.8
Female (n=243)	49	20.2	43	17.7
Age (years)				
18-25 (n=246)	44	17.9	33	13.4
26-35 (n=64)	18	28.1	12	18.8
36-45 (n=67)	12	17.9	15	22.4
46 and more (n=33)	3	9.1	6	18.2
Nationality				
Saudi (n=398)	75	18.8	64	16.1
Non-Saudi (n=12)	2	16.7	2	16.7
Level of Education				
Below secondary (n=25)	3	12.0	1	4.0
Secondary (n=91)	18	19.8	21	23.1
University (n=294)	56	19.0	44	15.0
State of job				
Students (n=236)	46	19.5	34	14.4
Not employed (n=74)	12	16.2	14	18.9
Employee (n=100)	19	19.0	18	18.0
Income				
Less than 5000 SAR (n=270)	52	19.3	44	16.3
More than 500 SAR (n=140)	25	17.9	22	15.7
Marital status				
Married (n=144)	27	18.8	25	17.4
Single (n=257)	48	18.7	36	14.0
Widow/Widower (n=4)	2	50.0	3	75
Divorced (n=5)	0	0	2	40.0
Residency				
Urban (n=208)	43	20.7	27	13.0
Rural (n=202)	34	16.8	39	19.3

## Discussion

The objective of this study was to assess the perception and understanding of psoriasis among the general population in Jazan, Saudi Arabia. The findings revealed that 79% of the respondents were familiar with the term "psoriasis" in Arabic, which is comparable to the awareness level reported in a German study (80%). However, it was observed that familiarity with the term did not necessarily correspond to accurate knowledge and awareness about the disease. Numerous misconceptions were identified among the participants, such as the belief that psoriasis does not significantly burden the affected individual (52% of participants) or their relatives (nearly 70% of participants). These findings contradict other published articles that demonstrate the impact of psoriasis on various aspects of quality of life, including social life, sports, home care, personal and sexual relationships, and work [16,18].

When the association between the social demographic variables was assessed, there was a statistically significant relationship between age and psoriasis (p < 0.001). Psoriasis prevalence increased with age, with people who were aged 46 and above having the highest occurrence. This suggests that psoriasis may be more likely to develop or be diagnosed in older individuals. This finding is also consistent with a study conducted in referral centers which found that psoriasis is affected by old age [21]. While the association between gender and psoriasis was not statistically significant (p = 0.113), the data showed a higher percentage of females knowing someone with psoriasis. This could reflect a potential gender difference in awareness or reporting [22]. Employment also was significantly associated with psoriasis (p = 0.018). Employed individuals and students reported higher rates of psoriasis, indicating potential stress-related factors in the development of the condition. The findings offer valuable insights into the potential risk factors and social determinants of the condition, highlighting the complex interplay between genetics, environmental factors, and lifestyle.

In addition, other social dimensions of this study focused on the public perception towards psoriasis patients. More than 70% of the respondents expressed sympathy towards people suffering from psoriasis. It shows that their community is empathetic thus offering encouragement to those people suffering from psoriasis. This sympathy expressed in most parts of the city by the majority presents a humane society. A vast majority (79.8%) felt that people who have psoriasis should receive better care. Recognizing that psoriasis patients require more support is imperative in improving their overall quality of life. About 61% of the participants would be ready to eat with psoriasis victims at the same table, 65.1% would shake hands with them, and as high as 85.1% are ready to live in the same household. These findings suggest that people with psoriasis enjoy a general acceptance, inclusiveness, and tolerant attitude. A similar study conducted by Ghorbanibirgani et al. among the Iranian population found that the majority of psoriasis patients face stigma and rejection [23]. The differences in these findings could be attributed to differences in geographical areas, cultural beliefs, and access to information.

The study further sought to find the public knowledge of the WHO resolution and World Psoriasis Day. It was found that a significant proportion of the surveyed population was informed about these global initiatives. Exactly, 18.8% of respondents knew about the WHO resolution, and 16.1% were informed about World Psoriasis Day. These proportions indicate a low level of awareness within the Jazan region. The results also show notable gender differences in awareness levels. While 20.2% of females knew about the WHO resolution, only 16.8% of males did. Similarly, regarding World Psoriasis Day, the proportion was higher among females (17.7%) than among males (13.8%). The gender differences could be caused by differences in information sources and healthcare-seeking behaviors between genders. A study by Guillet et al. agrees with our findings attributing higher awareness among females to their high susceptibility to the disease [24].

Discriminatory behaviors towards psoriatic patients, resulting from cultural misconceptions or stigmatization, were detected in previous studies [25,26]. In the present study, we identified behaviors such as participants avoiding sharing the same swimming pool, forming personal relationships, shaking hands, or living in the same household as a psoriasis patient. These responses indicate that a small percentage of the population still holds prejudices and inhibitions when it comes to interacting with psoriasis patients, emphasizing the necessity for further public information campaigns.

Limitations

However, it is important to acknowledge the limitations of this study, including its cross-sectional design, which may introduce recall and information bias. Additionally, the study was conducted solely in Jazan, Saudi Arabia, limiting the generalizability of the findings to other urban and suburban regions of the country. Simple random sampling could be adopted to ensure the sample is representative of the population of interest. Therefore, it is recommended to conduct a larger nationwide multicenter survey to encompass the diversity of the Saudi Arabian population.

Recommendations

We recommend that healthcare practitioners should make initiatives that promote public education and awareness campaigns to dispel misconceptions about psoriasis and its impact. The awareness campaigns should focus on promoting accurate information about the disease. Effective policies should be developed to enhance greater inclusivity and moral support for individuals with psoriasis, addressing the stigma and discrimination that they face. Finally, future research should put more emphasis on the cultural values and regional influences that promote misconceptions and stigma to develop tailored interventions to promote understanding and compassion toward psoriasis patients.

## Conclusions

In conclusion, this study offers important insights into the perception attitude and knowledge of psoriasis among the general population. The study revealed that a substantial proportion of the participants knew about psoriasis but also revealed a gap existed between awareness and practice. There were misconceptions about the burden of psoriasis on individuals and patients indicating the need for public education and awareness campaigns. The study found there were significant associations between psoriasis and various sociodemographic factors, including age, gender, and employment status, showing potential risk factors and social determinants of the disease. These findings reiterate that is important to combat stigma and foster a more inclusive and informed society in Jazan. Awareness initiatives targeting misconceptions and promoting empathy are crucial for improving the quality of life for psoriasis patients and creating a more compassionate community.
